# Age- and gender-related characteristics of astigmatism in a myopic population

**DOI:** 10.3389/fmed.2022.1011743

**Published:** 2022-10-13

**Authors:** Shan Yang, Yang Jiang, Ge Cui, Ying Li

**Affiliations:** Department of Ophthalmology, Peking Union Medical College Hospital, Chinese Academy of Medical Sciences & Peking Union Medical College, Beijing, China

**Keywords:** age, gender, astigmatism, cornea, myopia

## Abstract

**Purpose:**

To explore age- and gender-related differences of refractive and corneal astigmatism in myopic patients looking for refractive surgery.

**Design:**

A retrospective cross-sectional study.

**Materials and methods:**

The medical files of candidates looking for corneal refractive surgery between 2019 and 2021 were reviewed, demographic and refractive parameters including age, gender, refractive status, and corneal parameters were analyzed.

**Results:**

A total of 1,417 eyes of 1,417 patients (453 males and 964 females) were included. Males had thicker cornea than females, while females had steeper cornea than males, there was no gender-related difference in refractive and corneal astigmatism depending on patients’ age. There was no difference in refractive astigmatism among different age group from 18 to 50 years, while corneal astigmatism had a shift from with-the rule (WTR) to against-the-rule (ATR) with increasing age. Age, central corneal thickness (CCT), sphere, refractive astigmatism (RA), and corneal curvature (Km) were correlated with corneal astigmatism (CA) (standardized coefficients of are 0.006, *p* = 0.011 for age, −0.001, *p* = 0.004 for CCT, and −0.027, *p* < 0.001 for sphere, 0.61, *p* < 0.001 for RA, −0.05, *p* < 0.001 for corneal curvature).

**Conclusion:**

Refractive astigmatism is stable until the age of 50 years in myopic patients looking for refractive surgery, while corneal astigmatism showed a shift from WTR to ATR with advancing age. Age, CCT, sphere, refractive astigmatism and corneal curvature (Km) were correlated with corneal astigmatism.

## Introduction

Astigmatism is a common type of refractive error, which has a significant impact on visual performance. It was first described by Tomas Young in the early 1800s, since then, a large number of studies have been conducted to evaluate various aspects of astigmatism (1–3). However, there is no single model or theory can explain the genesis of astigmatism (4). With the surge in corneal refractive surgery and toric lens implantation, it’s important to know the characteristics of refractive and corneal astigmatism as it may provide important information in making guidelines and surgical design.

It’s known that the prevalence and distribution of corneal or refractive astigmatism change according to the ethnicity and study population, it also varied with age, gender, and refractive statues (5–7). Previous studies revealed that corneal shape and astigmatism change from with-the-rule astigmatism to against-the-rule astigmatism with increasing age (8). And the amount and axis of astigmatism differ between males and females (9). But most of the studies were conducted on older population, cataract patients, or a wide range of ages (10, 11).

To our knowledge, there is no published study investigated the age- and gender-related characteristics in Chinese young myopic patients. The aim of this study is to describe the age- and gender-related difference in refractive and corneal astigmatism in this population, and its association with other corneal parameters.

## Materials and methods

### Subjects

This study reviewed and analyzed data of patients looking for laser refractive surgery between 2019 and 2021. The inclusion criteria were: refraction power was stable for more than 2 years (myopia increase ≤−0.5D); and no ocular and systemic disease (pterygium, keratoconus, etc.). Eyes with a history of ocular surgery, fundus disease, or other abnormal corneal disease or opacity were excluded.

### Data collection

The medical files of patients were reviewed, demographic and preoperative data were collected including: age, gender, sphere, refractive astigmatism, flat K, steep K, corneal astigmatism, intraocular pressure, and central corneal thickness (CCT). Manifest and cycloplegic refraction were assessed by the same optometrist. Corneal curvature was obtained by corneal topography (Tomey, TMS-4, Japan), IOP was measured with a non-contact tonometer (Canon Full Auto Tonometer TX-F; Canon, Inc., Tokyo, Japan). CCT was measured with Sonomed Micropach 200P + (Sonomed, New Hyde Park, NY, USA). CCT, Manifest and cycloplegic refraction and corneal topography were performed by two experienced operators (L.S, YM. J). Date obtained from the right and left eye of a subject are often correlated, so the date of the right eye in this study was used for statistical analysis (12).

Refractive data were converted to minus cylinder form during analysis. The axis of astigmatism was defined for 180° ± 30° as WTR, for 90° ± 30° as ATR, whereas 45° ± 15° and 135° ± 15° were regarded as oblique astigmatism.

### Statistical analysis

Student *t*-test was used to compare the difference between male and female. One-way analysis of variance (ANOVA) was used for comparison among multiple groups. Multiple linear regression analyses were used to assess the contribution of age, gender, central corneal thickness, refractive error, and corneal curvature to the corneal astigmatism. *P* < 0.05 were considered statistically significant. Statistical analyses were performed using SPSS and Excel.

## Results

This study enrolled 1,417 eyes of 1,417 Chinese patients (453 males and 964 females) with a mean age of 36.03 ± 5.7 years (18∼50 years). The mean central corneal thickness (CCT) was 537.1 ± 33.27 μm, the mean intraocular pressure (IOP) was 16.13 ± 2.62 mmHg, the mean sphere power and astigmatism was −5.32 ± 2.58D, −0.88 ± 0.91D, the mean corneal power, and corneal astigmatism was 43.64 ± 1.49D, −1.21 ± 0.75D. The male eyes had greater CCT than female eyes (543.6 μm vs. 537.7 μm, *p* < 0.01), while females had slightly steeper corneal curvature (43.75 vs. 43.41, *p* < 0.01). There was no statistical sex-related difference in IOP, sphere, refractive astigmatism (RA), and corneal astigmatism (CA) as shown in Table 1.

**TABLE 1:** Ocular parameters of patients stratified by gender.

	Number	Age	CCT	IOP	Sphere	RA	Km	CA
Total	1417	36.03 ± 5.7	537.1 ± 33.27	16.13 ± 2.62	−5.32 ± 2.58	−0.88 ± 0.91	43.64 ± 1.49	−1.21 ± 0.75
Male	453	34.86 ± 5.8	543.6 ± 31.74	16.13 ± 2.69	−5.28 ± 2.63	−0.91 ± 0.91	43.41 ± 1.52	−1.23 ± 0.76
Female	964	36.59 ± 5.7	537.7 ± 33.78	16.07 ± 2.61	−5.46 ± 2.52	−0.85 ± 0.85	43.75 ± 1.47	−1.19 ± 0.74
*p*-value		**<0.001**	0.002	0.66	0.19	0.18	**<0.001**	0.45

CCT, central corneal thickness; IOP, intraocular pressure; RA, refractive astigmatism; Km, mean keratometry; CA, corneal astigmatism.

In the total population, 974 (68.73%) eyes had refractive astigmatism (RA) over 0.5D, the mean magnitude of RA was 0.88 ± 0.91D, 1,210 (85.39%) of all eyes had corneal astigmatism (CA) over 0.5D, the mean magnitude of CA was 1.21 ± 0.75D. The mean magnitude of RA and CA did not have a significant difference between the men and women groups in any age group. The magnitude of RA did not change significantly among age groups, while the CA in male eyes decreased significantly with advancing age (*p* = 0.008) (Table 2).

**TABLE 2:** Univariate comparison of the mean magnitude of refractive astigmatism and corneal astigmatism stratified by age and gender.

	Age group				*P*-value
	
		18∼29 (*N* = 191)	30∼39 (*N* = 837)	40∼49 (*N* = 389)	
Mean magnitude of RA	Male	1.01 ± 0.83	0.88 ± 0.89	0.85 ± 1.05	0.13
	Female	0.96 ± 0.89	0.86 ± 0.83	0.79 ± 0.86	0.2
*P*-value		0.31	0.77	0.55	
Mean magnitude of CA	Male	1.45 ± 0.76	1.18 ± 0.72	1.17 ± 0.83	0.008
	Female	1.32 ± 0.67	1.22 ± 0.73	1.13 ± 0.79	0.051
*P*-value		0.22	0.48	0.62	

RA, refractive astigmatism; CA, corneal astigmatism.

**FIGURE 1:**
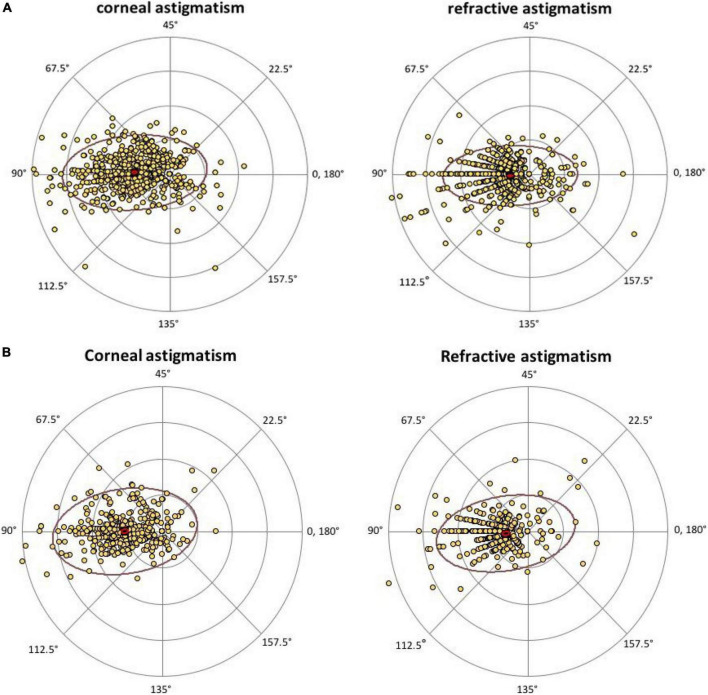
Double-angle plot of refractive astigmatism and corneal astigmatism in male **(A)** and female **(B)**. Data are scattered mostly to the left of the center, indicating that RA and CA tend to have a with-the-rule pattern. RA, refractive astigmatism; CA, corneal astigmatism.

Most cases had with-the rule (WTR) astigmatism, followed by against-the rule (ATR) and oblique. Regarding to refractive astigmatism, 878 (88.15%) eyes had WTR astigmatism, 84 (8.43%) eyes had ATR astigmatism, 36 (3.61%) eyes had oblique astigmatism. The distribution was similar in corneal astigmatism, in which 1,203 (86.92%) eyes had WTR astigmatism, 99 eyes (7.15%) had ATR astigmatism, and 82 eyes (5.92%) had oblique astigmatism. Figure 1 are double-angle plots of RA and CA, data are scattered mostly to the left of the center, indicating that RA and CA tend to have a with the rule pattern.

**FIGURE 2:**
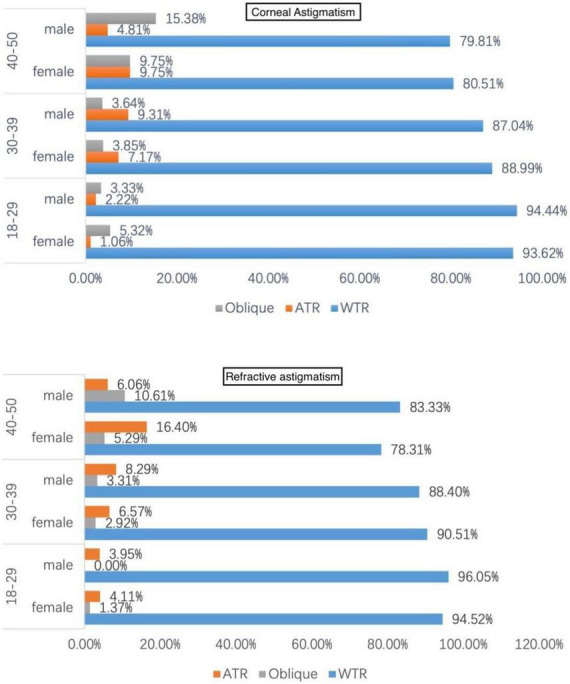
Distribution of astigmatism axis stratified by gender and age. WTR, with-the-rule; ATR, against-the-rule.

For the axis of refractive astigmatism and corneal astigmatism, there was no significant difference between men and women eyes in the percentage of WTR, ATR, and oblique at any age group. While the percentage of WTR, ATR, and oblique astigmatism was significantly different among different age groups, there was a shift in astigmatism from WTR to ATR with advancing age (Figure 2).

The correlations between corneal astigmatism and demographic and ocular parameters were analyzed by multivariate linear regression. Age, CCT, sphere, RA, and corneal curvature (Km) were correlated with CA (standardized coefficients of are 0.006, *p* = 0.011 for age, −0.001, *p* = 0.004 for CCT, and −0.027, *p* < 0.001 for sphere, 0.61, *p* < 0.001 for TA, −0.05, *p* < 0.001 for mean corneal curvature), as shown in Table 3. RA had the most significant correlation with CA, followed by corneal curvature, sphere, age, and CCT.

**TABLE 3:** Multiple linear regression model estimating the correlation between age, gender, central corneal thickness, sphere, corneal curvature, and corneal astigmatism.

	Unstandardized coefficients (β)	Standard error (SE)	Standardized coefficients (β)	*t*	*P*-value
Constant	1.749	0.471		3.714	< 0.001
Age	0.006	0.002	0.046	2.56	0.011
Gender	–0.011	0.029	–0.007	–0.384	0.701
CCT	–0.001	0.000	–0.051	–2.891	0.004
Sphere	–0.027	0.005	–0.092	–5.132	< 0.001
TA	0.61	0.15	0.726	40.211	< 0.001
Km	–0.05	0.009	–0.099	–5.432	< 0.001

CCT, central corneal thickness; TA, total refractive astigmatism; Km, mean corneal curvature.

## Discussion

Sex difference exists in a wide range of the eye. For instance, a large number of studies found that males have different axil length and anterior chamber depth with females (5, 13, 14). In this study, we found that males had thicker corneal thickness than females, and females had steeper corneal curvature than males, Wang’s study also represented the same results (15). Therefore, it’s of importance to take sex effect into consideration when setting up the eye’s “average” values.

Regarding the myopic population, previous literature has focused on the characteristics of astigmatism and its correlation with other ocular parameters, while differences in age and sex of myopic populations have been rarely reported (16, 17). The prevalence of refractive astigmatism in our study was 68.73%, one study conducted in school students of eastern China found the prevalence of refractive astigmatism was 7∼25% and increased with grade (18). The prevalence of corneal astigmatism in our study was 85.39%. Other studies reported the prevalence of corneal astigmatism was around 60% (10, 19), this inconsistency was due to the definition of astigmatism used and the age and ethnicity of population examined. Although previous studies described a sex-related difference for the magnitude of astigmatism, this wasn’t shown in our study. But those studies were conducted in European (20) or African population (21), other studies in Asian population didn’t observe the sex difference either (22, 23).

Regarding to age-related difference, the magnitude of total refractive astigmatism did not appear to be age dependent in our study, while the magnitude of corneal astigmatism decreased with age, previous studies also found that patients age was negatively correlated with the magnitude of corneal astigmatism (9, 24, 25). One explanation is that the intraocular astigmatism increased with age, which comprise the effect of corneal astigmatism on the total refractive astigmatism. The age-related changes in the axis of the refractive and corneal astigmatism have been described in several studies (26–28), they found that there was a shift in astigmatism from with-the-rule to against-the-rule with increase in age, which was also presented in our study. Although the exact mechanism of astigmatism is still unknown, this age-related change is thought to be related to changes in the collagen orientation and the position and extension of the lid, and extraocular muscles (4, 29, 30).

It’s well known that corneal astigmatism is the leading cause of refractive astigmatism. In our study, we found that corneal astigmatism was strongly positive correlated with refractive astigmatism, which was consistent with previous studies (31). We also found that central corneal thickness and corneal curvature have a weak but significant correlation with corneal astigmatism. This relationship between corneal astigmatism and corneal thickness and corneal curvature was rarely reported before. Yuta Ueno and associates found that the difference between horizontal and vertical corneal thickness was correlated with the posterior corneal astigmatism (32), Zhou X etc., discovered that the front and back curvatures and the back astigmatism correlate with the distribution of corneal thickness (33). In addition, we also found that myopia is associated with increased astigmatism, which was also found in a large German population-based study (34).

There are several limitations in our study. First, we are unable to measure intraocular astigmatism, therefore, we were not able to describe the changes of posterior cornea or lens astigmatism. Second, our data only included cases with an age range from 18 to 50 years, therefore we can’t describe characteristics of astigmatism during childhood or the elderly population. Third, we didn’t include anterior chamber depth and axial length in the association analysis of astigmatism, which needs further investigation.

## Conclusion

In summary, our study found that there was a sex-related difference in corneal thickness and corneal power, but not in refractive and corneal astigmatism in young adults. In addition, corneal astigmatism was associated with age, corneal thickness, corneal power, and spherical refraction. We confirmed that there was a change from with-the-rule astigmatism to against-the-rule astigmatism in young adults.

## Data availability statement

The raw data supporting the conclusions of this article will be made available by the authors, without undue reservation.

## Ethics statement

The studies involving human participants were reviewed and approved by Peking Union Medical College Hospital. The patients/participants provided their written informed consent to participate in this study.

## Author contributions

All authors listed have made a substantial, direct, and intellectual contribution to the work, and approved it for publication.
